# P24 TB or not TB? A case of severe multi-organ erosive rheumatoid arthritis in a Ukrainian refugee

**DOI:** 10.1093/rap/rkad070.045

**Published:** 2023-09-27

**Authors:** Hannah Cooke, Iulia Biliavska, Emma M Clark, Benjamin G Faber

**Affiliations:** Musgrove Park Hospital, Taunton, United Kingdom; Southmead Hospital, Bristol, United Kingdom; Southmead Hospital, Bristol, United Kingdom; Southmead Hospital, Bristol, United Kingdom

## Abstract

**Introduction:**

Since the onset of the conflict in Ukraine in February 2022, the government has helped over 220,000 Ukrainians settle in the United Kingdom as refugees. This rapid increase in the number of displaced Ukrainian citizens raises the importance of understanding the health seeking behaviours, disease treatment, and prevalence of rheumatological conditions and infectious diseases in this cohort.

Herein, we present the case of a Ukrainian refugee with rheumatoid arthritis and radiological evidence of pulmonary nodules to highlight the challenges and considerations for the rheumatologist in this context.

**Case description:**

A 73-year-old Caucasian woman was displaced from Ukraine to the United Kingdom as a refugee in early 2023. She had been diagnosed with rheumatoid arthritis in 1983 and managed in Ukraine until her first contact with NHS rheumatology services after settling in Bristol.

At presentation in the UK, her main concerns in clinic were lethargy, knee pain, sicca symptoms and occasional fevers but no respiratory symptoms. She was being managed on a combination of oral methylprednisolone, diclofenac and hyaluronic acid eye drops, with no conventional disease modifying treatment. Translation was facilitated by her daughter, who explained that previous trials of methotrexate had not been tolerated due to gastrointestinal side effects. The patient had been encouraged to try further disease modifying treatment but had declined.

Examination revealed extensive joint deformity and widespread rheumatoid nodules. Strikingly, she had notable subluxation throughout her hands and feet, and varus deformity of both knees. Hand X-rays confirmed extensive erosive changes including subluxation of her metacarpophalangeal with ulnar deviation and fixed flexion deformities of her proximal interphalangeal joints. Knee X-rays also demonstrated complete loss of her medial joint space and widespread osteophytosis.

Blood tests demonstrated strongly positive rheumatoid factor (116.8 IU/ml) and anti-cyclic citrullinated peptide (>250 units/ml); anti-Ro, anti-La, and anti-Ro52 were also positive. Inflammatory markers were mildly elevated (C-reactive protein 10 mg/L and plasma viscosity 2.10 mPas).

Chest X-ray demonstrated diffuse widespread ∼3-4cm opacifications in keeping with lung nodules. Interferon gamma release assay testing was indeterminate. Chest CT characterised some nodules as indeterminate due to calcification. Further microbial testing and imaging are awaited.

The patient was commenced on sulfasalazine, switched to oral prednisolone and referred to a lung multi-disciplinary meeting, for a bone health assessment and ophthalmology review, and will have specialist nurse and physiotherapy input. Specialist rheumatology psychological input is planned.

**Discussion:**

This case highlights a severe case of rheumatoid arthritis and Sjögren’s syndrome with multi-organ disease which were further compounded by the additional challenges that arise in the management of patients with refugee status. Not only may there be a more severe manifestation of disease due to interruptions in treatment or lack of access to specialist care, but there must also be an awareness of the psychological and emotional impact that displacement from a war zone can cause.

A specific consideration in this patient is the importance of evaluating for underlying TB as a differential cause of lethargy, lung lesions, and fever. This is particularly of relevance as Ukraine has the fourth highest incidence of tuberculosis in the WHO European region. Access to, and delivery of, medications for TB have exacerbated this since the onset of the conflict.

Further to this, embedding such patients within NHS rheumatology services can prove challenging. Often there is a lack of access to past medical records and summaries of previous investigation and treatment. In our patient they were unaware of any previous lung investigations or diagnoses. Standard management of rheumatoid arthritis in Ukraine includes the widespread use of disease modifying agents. However, if patients are unable or unwilling to engage with services, they may go on to develop erosive disease from lack of appropriate treatment. As in this case, it is important to explore the reasoning behind this, and to have an awareness that introducing change to long term management may be met with trepidation.

Language barriers pose challenges to communication and, although translation can be facilitated in some cases by relatives, often there is a need to involve third party interpreting services. Within our team, the presence of a Ukrainian healthcare professional was highly beneficial for both understanding and reassurance for the patient.

**Key learning points:**

There are several learning points that can be derived from this case. Firstly, due to advances in the management of rheumatoid arthritis, such severe erosive disease is thankfully rarely seen in the UK. However, due to a combination of factors, this patient developed disabling joint destruction as well as secondary Sjögren’s and lung involvement with nodules. It is important to recognise that patients displaced from other regions may have a more severe manifestation of rheumatoid arthritis than those diagnosed and treated within the UK and may therefore need more intensive follow-up and multi-disciplinary input.

Secondly, it is important to assess patients with a nodular appearance to chest X-ray for underlying tuberculosis and malignancy. This is particularly important in the context of patients displaced from areas of higher TB prevalence, and may have a significant impact on their management.

Finally, the increase in refugee patients from Ukraine emphasises the need for increased understanding of their healthcare needs. Social and communication barriers can exacerbate challenges with management. This extends beyond the management of physical illness but should also include a holistic approach to management of psychological distress and disability and access needs.

